# The Effect of Cold Showering on Health and Work: A Randomized Controlled Trial

**DOI:** 10.1371/journal.pone.0161749

**Published:** 2016-09-15

**Authors:** Geert A. Buijze, Inger N. Sierevelt, Bas C. J. M. van der Heijden, Marcel G. Dijkgraaf, Monique H. W. Frings-Dresen

**Affiliations:** 1 Department of Orthopaedic Surgery, Academic Medical Center, Amsterdam, The Netherlands; 2 Department of Orthopaedic Surgery, Medical Center Slotervaart, Amsterdam, The Netherlands; 3 Risk Management, Achmea, Zeist, The Netherlands; 4 Clinical Research Unit, Academic Medical Center, Amsterdam, The Netherlands; 5 Department Coronel Institute of Occupational Health, Academic Medical Center, Amsterdam, The Netherlands; TNO, NETHERLANDS

## Abstract

**Purpose:**

The aim of this study was to determine the cumulative effect of a routine (hot-to-) cold shower on sickness, quality of life and work productivity.

**Methods:**

Between January and March 2015, 3018 participants between 18 and 65 years without severe comorbidity and no routine experience of cold showering were randomized (1:1:1:1) to a (hot-to-) cold shower for 30, 60, 90 seconds or a control group during 30 consecutive days followed by 60 days of showering cold at their own discretion for the intervention groups. The primary outcome was illness days and related sickness absence from work. Secondary outcomes were quality of life, work productivity, anxiety, thermal sensation and adverse reactions.

**Results:**

79% of participants in the interventions groups completed the 30 consecutive days protocol. A negative binomial regression model showed a 29% reduction in sickness absence for (hot-to-) cold shower regimen compared to the control group (incident rate ratio: 0.71, P = 0.003). For illness days there was no significant group effect. No related serious advents events were reported.

**Conclusion:**

A routine (hot-to-) cold shower resulted in a statistical reduction of self-reported sickness absence but not illness days in adults without severe comorbidity.

**Trial Registration:**

Netherlands National Trial Register NTR5183

## Introduction

Cold bathing is a common custom in many parts of the world. Ever since the introduction of civilized bathing, humans have experimented with water temperature variation to expose the body to extreme conditions. In ancient times, Roman bathing was based around the practice of moving through a series of heated rooms culminating in a cold plunge at the end.[1] In modern times, the traditional ritual of the *frigidarium* has been kept in most saunas and spas around the world.

Cold bathing has been claimed to have multiple beneficial effects on health such as improvement of the immune system, cardiovascular circulation and vitality, but any true association remains unclear.[2] Previous investigations on the short-term effects of cold exposure have shown increases of cortisol and norepinephrine concentrations with modulation of the physiological response but showed minimal or no immune modulation.[3–7] However, the cumulative clinical effect and relevance for health after adaption of cold exposure (response conditioning) in healthy humans remain speculative as randomized controlled trials are lacking.

The primary objective of this trial was to determine whether perceived illness could be modulated after repeated pragmatic cold exposure by taking a cold shower for at least 30 consecutive days. Secondary objectives were to determine whether there was any effect on quality of life, work productivity and anxiety as well as adverse reactions. A dose response relationship was investigated by varying in the duration of the cold shower.

## Methods

### Study Design

This parallel group, unblinded, randomized controlled trial was designed following CONSORT guidelines and took place in The Netherlands, named the Cool Challenge. Between December 7^th^ and December 30^th^ 2014, we recruited participants through advertisements and (social) media. Inclusion, randomization and data collection were all performed via a web based application using surveys only. Written informed consent was obtained from all participants. The study was designed as a pragmatic trial and compliance to the intervention could not be verified. The primary aim was to look at any effect of a routine cold shower and the secondary aim was to look at dose-dependency effects. The study protocol was approved by our institutional review board based on ethical considerations (September 3^rd^ 2014, Academic Medical Center, Amsterdam, The Netherlands). Being exempt from formal medical ethical review as it was considered non-medical research, this non-clinical trial was not registered in a clinical trial registry before recruitment of the first participant but on June 25th 2015 prior to data analysis (August 5^th^—September 13^th^ 2015) with The Netherlands National Trial Register (NTR), approved by the WHO, number NTR5183. The authors confirm that all ongoing and related trials for this intervention are registered.

### Participants

Participants were adults aged 18–65 without routine experience of (hot-to-) cold showering who were employed when they entered the study. As no harmful effects of cold showering have previously been reported, the only exclusion criterion was significant comorbidity, including cardiac, pulmonary or any other severe disease. Exclusion criteria were primarily self-assessed. Significant comorbidity was defined by either a subject’s positive answer to the self-assessment question: “Do you have a severe medical condition to the heart or lungs?” or at investigator’s judgement of the subject’s self-reported medical conditions. Subjects were asked to answer the question: “Do you have a medical condition?” Careful screening was done for any severe cardiac, pulmonary or other systemic comorbidity at the investigator’s judgement. After informed consent, eligible participants were randomized to one of four groups (1:1:1:1). Randomization was performed using computerized random numbers within a custom-made Hypertext Preprocessor (PHP) scripted web-based application for online surveys, without applying block randomization or stratification methods. The function RAND with PHP programming language was used, which assigns a random number between 1 and 4 each time.[8] Allocation concealment was ensured within the web-based application.

### Procedures

Participants randomized to the intervention groups were instructed to shower as warm and as long as preferred but ending with respectively 30, 60 or 90 seconds showering at the coldest available water temperature. They were instructed to use either the timer provided through a web link for smart phones by the research team, or a timer of their own. In case they could not complete the full period, participants were asked to time the period using a stopwatch. The average ground temperature at the level of running water in The Netherlands was 10°C during the study period with average cold water temperatures of 10–12°C.[9] The intervention period was 30 consecutive days from January 1^st^-30^th^ 2015. During the following 60 days January 31^st^-March 31^st^ 2015 participants of all three intervention groups were instructed to shower as preferred, i.e. taking cold showers as often and as long as preferred. Participants randomized to the control group were instructed to shower as regular (not cold) during the full 90-day study period.

Data were primarily collected through an online web-based platform and managed in Microsoft Excel 15.0 (Microsoft Corporation, Redmond, Washington, USA). In order to provide self-reports, participants were asked to log in three times: at baseline, between 30 and 60 days, and between 90 and 120 days. Weekly reminder emails were sent to participants who had not yet completed follow-up. Specific missing data were collected by email.

### Outcomes

All outcomes were self-reported using web based surveys. The primary outcome was illness days and related leave from work during the 90-day study period (January to March 2015). Sickness absence was considered to be the most objective indirect parameter indicative of illness severity. Participants were asked to rate the total number of days of absence from their work due to sickness, if possible by verifying with their employer or their agenda. Absence frequency was not measured. Illness days were defined as the total amount of days a participant felt ill (including symptoms of cold and flu). Participants were asked to rate the number of days that they had “symptoms of illness, cold or flu” during the study period. If participants rated sickness absence or illness over five days, they were asked for a reason. The secondary outcomes were time of subjective sickness, quality of life, work productivity, thermal sensation and anxiety.

- Quality of life was assessed using the Short Form 36 (SF-36), a 36-item patient-reported survey of health.[10] Results were analyzed including the physical component summary (PCS) and mental component summary (MCS), with higher scores representing better quality of life. The Dutch 4-week recall version was used, as adapted and validated by Aaronson et al.[11]
- Work productivity was assessed using the Utrecht Work Engagement Scale (UWES), a 9-item survey with a 7-point Likert scale of engagement during work activities (0 = “never” to 6 = “every day”) and the total sum scores (range 0–54) were used for analysis, with higher scores representing better work productivity. [12–13]
- Mediation of the mind-body neurosensory pathways were assessed as changes in daily thermal sensation of the body and extremities, respectively (expressed as warmer, colder or not different from their habitual daily sensation before the start of the trial).
- To explore anxiety, we selected the six questions of the subscale scoring anxiety from the Brief Symptom Inventory.[14] The total anxiety score (range 0–24) of this self-report inventory with a 5-point Likert scale of distress (0 = "not at all" to 4 = "extremely") was used for analysis with lower scores representing less anxiety. The Dutch version was used, as adapted and validated by De Beurs and Zitman.[15]
- At each follow-up moment, participants were asked to report any positive and negative effects. Adverse reactions other than influenza or influenza-related symptoms that were possibly or likely related to (hot-to-)cold showering were recorded by asking participants for any negative experiences and events as well as reasons to discontinue the intervention.

### Statistical Analysis

We calculated that 575 individuals were required to achieve 80% power to detect a difference of 0.5 days of sickness absence (SD 3.03) during the 90 days period, based on previous data.[16] The significance level (alpha) of the test was set at P<0.05. Accounting for a 20% lost to follow-up, we set target enrolment at 720 individuals for each intervention group and the control group. We collected participants’ characteristics and baseline values for primary and secondary outcome measures to allow comparisons between groups at baseline (Table 1). Analysis was conducted using intention to treat principles. For the primary outcome (sickness absence days and illness days at 90 days follow-up) a negative binomial regression model with log link was performed which was preferred over the Poisson model because of over-dispersion in the count data. The following parameters were tested by an analysis of deviance: group, age, sex, body mass index, regular physical activity (y/n), and fulltime employee (y/n). Statistical methods used 2-sided testing. For secondary outcomes this included Kruskal Wallis or Mann-Whitney U tests for non-normal distributed continuous variables and Chi-square tests for categorical variables. The level of significance of the primary outcome was set at P<0.05. For secondary outcome variables a Bonferroni adjustment was used to correct for multiple outcome variable testing, with the level of significance set at P*<*0.005. Analyses were performed with SPSS 23 (IBM SPSS, Chicago, USA) and SAS 9.4 (SAS Institute Inc., Cary, USA).

**Table 1:** Baseline characteristics, according to study group.

Charachteristics	30s Group (n = 798)	60s Group (n = 727)	90s Group (n = 775)	Control Group (n = 718)
Women	473 (59)	423 (58)	466 (60)	399 (56)
Mean (SD) age (years)	39.7 (11.3)	38.9 (10.6)	39.6 (10.6)	39.2 (10.6)
Mean (SD) body mass index (kg/m2)	23.7 (3.4)	23.9 (3.7)	23.6 (3.3)	23.9 (3.4)
Good subjective health	770 (96)	694 (95)	752 (97)	684 (95)
Median (interquartile range) SF-36 physical component score	84.2 (77.2–89.2)	84.2 (76.2–90.2)	85.2 (77.2–90.4)	84.2 (77.3–90.2)
Median (interquartile range) SF-36 mental component score	81.4 (69.8–87.6)	81.3 (69.7–88.2)	81.1 (69.7–88.8)	81.9 (69.6–88.6)
Median (interquartile range) work engagement score	41 (33–46)	41 (32–45)	41 (32–46)	41 (32–46)
Median (interquartile range) anxiety score	1 (3)	1 (3)	1 (3)	1 (3)
Regular physical activity	661 (83)	600 (83)	664 (86)	614 (86)
Fulltime employee	315 (39)	283 (39)	279 (36)	269 (37)
Residence conditions				
Single	207 (26)	196 (27)	171 (22)	190 (26)
Living with partner	237 (30)	206 (28)	224 (29)	209 (30)
Living with (partner and) children	354 (44)	325 (45)	380 (49)	319 (44)

This study investigated the effect of cold showering on health and work: a trial randomizing a (hot-to-) cold shower for 30, 60, 90 seconds or a control group during 30 consecutive days followed by 60 days of showering cold at their own discretion for the intervention groups. Values are numbers (percentages) unless stated otherwise

## Results

**Fig 1:**
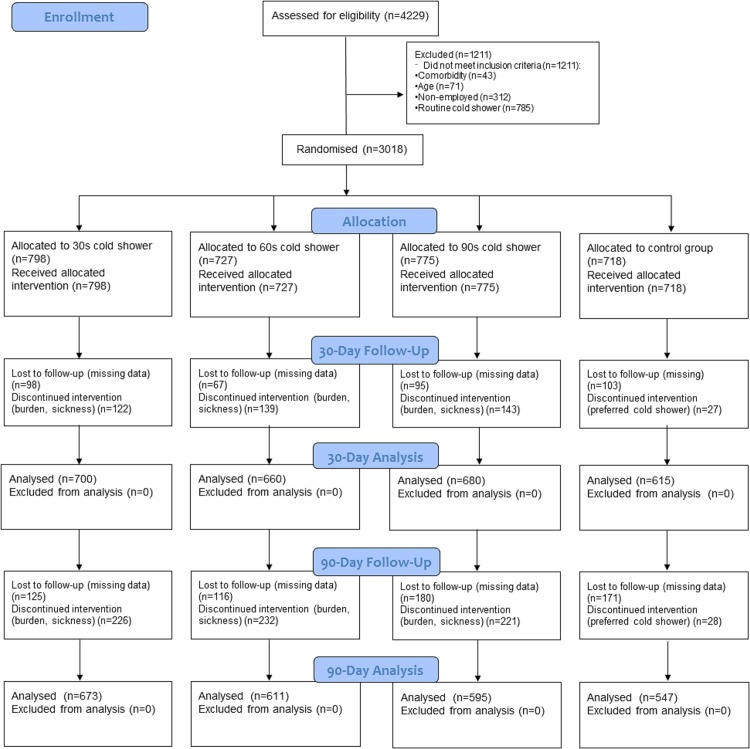
Study flow diagram.

Of the 4229 candidates screened for eligibility 3018 participants were enrolled (Fig 1). Loss to follow-up was 12% after 30 days, and 19.6% after 90 days. Table 1 shows that baseline characteristics as well as data on primary and secondary outcome measures were similar between the intervention groups and the control group. Results in text and tables are reported in respective order of the groups as 30s cold shower, 60s cold shower, 90s cold shower and control group. For the primary outcome sickness absence the individual cold shower regimes all differed statistically significant from the hot shower regimen (for 30s, 60s, and 90s: p = 0.014, p = 0.0268, and p = 0.0065, respectively). Analysis of deviance showed no statistically significant effect between the three cold shower groups (p = 0.98 for sickness absence, S1 Table). For illness days only the 60s cold shower regime differed statistically significant from the hot shower regimen: (for 30s, 60s, and 90s: p = 0.235, p = 0.014, and p = 0.383, respectively). Analysis of deviance showed no statistically significant effect between the three cold shower groups (p = 0.15 analysis of deviance, S2 Table). There were no trends between doses towards illness or absenteeism benefit.

Seventy-nine percent of participants in the interventions groups completed the 30 consecutive days protocol (82% *vs* 79% *vs* 79%; P = 0.14) and 64% continued the (hot-to-) cold shower on regular basis (66% *vs* 63% *vs* 62%; P = 0.36). A negative binomial regression model showed a 29% reduction in sickness absence for the (hot-to-) cold shower regimen compared to the control group (Incidence Rate Ratio (IRR): 0.71, P = 0.003). No significant difference between the intervention groups (P = 0.992) was observed, therefore parameter group was transformed into a factor with two levels, all intervention groups versus control group (Table 2). The only associated parameter of influence in the model was regular physical activity (IRR: 0.65, P = 0.003), which reduces the sickness absence by 35%. For illness days at 90 days follow-up there was no significant group effect, only a gender effect, with males showing a 14% reduction compared to females (IRR: 0.86, P = 0.010).

**Table 2:** Negative binomial regression model of the primary outcome.

Outcome	Median (interquartile range) per group	Range [Min, Max]	Percentage with any sickness respectively illness	Parameter	Maximum Likelhood Estimate (95% CI)	Exponential Estimate (95% CI)	P value
90 days sickness absence	30s Group: 0 (0–1)	[0, 62]	29,4%	Intercept	0.80 (0.49, 1.11)	2.23 (1.63, 3.03)	< .0001
	60s Group: 0 (0–1)	[0,29]	34,0%	Group (intervention groups *vs* control)*	-0.35 (-0.58, -0.12)	0.71 (0.56, 0.89)	0.003
	90s Group: 0 (0–1,5)	[0,40]	33,1%	Regular physical activity (yes *vs* no)*	-0.42 (-0.70, -0.15)	0.65 (0.5, 0.86)	0.003
	Control Group: 0 (0–2)	[0,51]	34,8%	Dispersion	4.64 (4.17, 5.15)		
90 days illness	30s Group: 2 (0–7)	[0,56]	65,0%	Intercept	1.27 (1.14, 1.39)	3.55 (3.13, 4.02)	< .0001
	60s Group: 2 (0–6)	[0,60]	63,3%	Group (intervention groups *vs* control)*	-0.12 (-0.26, 0.01)	0.89 (0.77, 1.01)	0.073
	90s Group: 2 (0–6)	[0,70]	64,5%	Gender (Male *vs* Female)*	-0.15 (-0.27, -0.04)	0.86 (0.76, 0.96)	0.0097
	Control Group: 2 (0–7)	[0,90]	69,3%	Dispersion	1.53 (1.41, 1.66)		

This study investigated the effect of cold showering on health and work: a trial randomizing a (hot-to-) cold shower for 30, 60, 90 seconds or a control group during 30 consecutive days followed by 60 days of showering cold at their own discretion for the intervention groups.

*The exponential of the estimates are Incident Rate Ratios (IRR)

Median quality of life MCS after 30 days was slightly higher for all intervention groups (84.7, interquartile range 76.4–90.2 *v* 85.1, interquartile range 76.7–90.6 *v* 85.7, interquartile range 78–90.8) compared to the control group (83.9, interquartile range 72.9–89.4) (Table 3). However, after 90 days significant differences were not observed anymore (Table 4). None of the other secondary outcomes were significantly different between groups at 30 and 90 days follow-up (Tables 3 and 4).

**Table 3:** Secondary outcomes at 30 days. Values are numbers (percentages) unless stated otherwise.

Outcomes		30s Group (n = 700)	60s Group (n = 660)	90s Group (n = 680)	Control Group (n = 615)	Group difference P value#	Intervention/control difference P value##
Median (interquartile range) sickness absence (days)		0 (0)	0 (0)	0 (0)	0 (0)	0.544	0.648
Median (interquartile range) illness (days)		0 (0–3)	0 (0–3)	0 (0–3)	1 (0–4)	0.232	0.047
Completed (hot-to) cold shower protocol during first 30 days*		573 (82)	513 (79)	530 (79)	N.A.	0.138	
Will to continue (hot-to) cold shower after first 30 days**		634 (93)	571 (89)	609 (91)	N.A.	0.024	
Median (interquartile range) SF-36 physical component score		86.2 (78.8–91.4)	87.2 (80.5–91.2)	87.2 (79.8–91.4)	85.4 (77.8–90.4)	0.017	0.006
Median (interquartile range) SF-36 mental component score		84.7 (76.4–90.2)	85.1 (76.7–90.6)	85.7 (78–90.8)	83.9 (72.9–89.4)	0.003	0.001
Median (interquartile range) work engagement score		42 (33–46)	42 (33–46)	42 (34–47)	40 (32–46)	0.108	0.020
Median (interquartile range) anxiety score		1 (0–3)	1 (0–3)	1 (0–3)	1 (0–3)	0.003	0.001
Thermal body sensation**						0.160	
	Warmer	262 (39)	265 (41)	269 (40)	N.A.		
	Colder	55 (8)	72 (11)	69 (10)	N.A.		
	No difference	363 (53)	304 (48)	333 (50)	N.A.		
Thermal hands and feet sensation**						0.778	
	Warmer	179 (26)	170 (26)	180 (27)	N.A.		
	Colder	79 (12)	88 (14)	90 (13)	N.A.		
	No difference	422 (62)	383 (60)	401 (60)	N.A.		

This study investigated the effect of cold showering on health and work: a trial randomizing a (hot-to-) cold shower for 30, 60, 90 seconds or a control group during 30 consecutive days followed by 60 days of showering cold at their own discretion for the intervention groups.

N.A. Not applicable

* Missing data in 5, 8, 7 participants (respectively)

** Missing data in 20, 19, and 9 participants (respectively)

# Difference between all groups (Kruskal Wallis)

## Difference between all interventional groups versus control group (Mann-Whitney U)

**Table 4:** Primary and secondary outcomes at 90 days. Values are numbers (percentages) unless stated otherwise.

Outcomes		30s Group (n = 673)	60s Group (n = 611)	90s Group (n = 595)	Control Group (n = 547)	Group difference P value#	Intervention/control difference P value##
Continued (hot-to) cold shower after first 30 days*		446 (66)	378 (63)	363 (62)	N.A.	0.355	N.A.
Median (interquartile range) frequency of cold shower (times per week)*		3 (0–7)	3 (0–7)	2 (0–6)	N.A.	0.727	N.A.
Median (interquartile range) duration of cold shower (s)*		30 (10–50)	60 (40–80)	60 (10–110)	N.A.	<0.001	N.A.
Will to continue (hot-to) cold shower after 90 days**		546 (88)	487 (84)	490 (85)	N.A.	0.199	N.A.
Median (interquartile range) SF-36 physical component score		85.8 (78.9–90.4)	86.4 (79.4–92)	87.2 (79.8–92)	86.4 (78.5–91.4)	0.121	0.338
Median (interquartile range) SF-36 mental component score		84.8 (76.7–89.6)	84.4 (75.7–90.2)	85.8 (78.0–90.6)	84.4 (74.3–90)	0.108	0.090
Median (interquartile range) work engagement score		41 (33–46)	42 (32–46)	42 (32–46)	41 (31.3–46)	0.638	0.389
Median (interquartile range) anxiety score		1 (0–3)	1 (0–3)	1 (0–3)	1 (0–3)	0.190	0.133
Reason of sickness absence if longer than 5 days***						0.326	
	Influenza	27 (64)	17 (46)	13 (42)	20 (51)		
	Psychosocial (including burnout)	6 (14)	7 (19)	6 (19)	5 (13)		
	Musculoskeletal Injury	4 (10)	4 (11)	2 (6)	3 (8)		
	Bronchitis/pneumonia	3 (7)	0 (0)	3 (10)	2 (5)		
	Other upper respiratory tract infection (excluding influenza)	2 (5)	0 (0)	2 (6)	2 (5)		
	Other infection(s)	0 (0)	5 (14)	1 (3)	3 (8)		
	Other comorbidity (including operation)	0 (0)	4 (11)	4 (13)	4 (10)		

This study investigated the effect of cold showering on health and work: a trial randomizing a (hot-to-) cold shower for 30, 60, 90 seconds or a control group during 30 consecutive days followed by 60 days of showering cold at their own discretion for the intervention groups.

N.A. Not applicable

* Missing data in 2 participants in 30s Group, and 2 participants in 60s Group

** Missing data in 56, 30, and 20 participants (respectively)

*** Data collected in 42, 37, 31, and 39 participants (respectively)

# Difference between all groups (Kruskal Wallis)

## Difference between all interventional groups versus control group (Mann-Whitney U)

Twenty serious adverse events were reported, that were all considered unrelated to the intervention. One participant in the 90 seconds intervention group died unexpectedly of occult chronic pulmonary embolism at 56 days follow-up. This occult condition was not diagnosed at the time of enrolment and her medical history included hypertension only. Critical assessment by the team of treating (intensive care) physicians showed no possible relationship to the (hot-to-)cold shower. There were eight participants with a mild pneumonia, two urinary tract infections, two had middle ear infections, one pneumothorax, one glaucoma, two hand wounds, one with multiple rib contusions after a fall, one with concussion and head wound after a fall, one bike and one ski-accident both with multiple minor contusions, distorsions and lacerations. No related serious adverse events were reported. The most common related mild adverse event was a persistent cold sensation after the cold shower in the body (196 participants) as well as in the hands and/or feet (257 participants), specifically in 3 participants with Raynaud’s phenomenon. Other possibly related adverse events included muscle ache or cramps in eight, itch in six, insomnia in four (related to cold shower in the evening), dizziness in four, lumbago in two, head ache in one, nose bleeding in one, diarrhea in one, palpitations in one and transient swelling and erythema of three digits of one hand in one participant after the cold shower.

## Discussion

In this pragmatic randomized controlled trial, routinely showering (hot-to-) cold resulted in a 29% reduction of self-reported sick leave from work but not illness days at 90 days follow-up in adults without severe comorbidity. The contrast between the results of both primary outcome parameters is suggestive of the fact that the intensity rather than the duration of symptoms is modulated by the intervention. Regular physical activity resulted in a 35% reduction of sickness absence. The combination of routine (hot-to-) cold shower and regular physical activity resulted in an expected 54% reduction of sickness absence compared to people who don’t do either. The duration of the cold shower did not influence outcome as there was no significant difference between intervention groups. The only secondary outcome that showed a slight beneficial effect–on the short run–was quality of life (mental component summary) although this was deemed too small to be clinically relevant. Even though the vast majority of participants reported a variable degree of discomfort during cold exposure, the fact that 91% of participants reported the will to continue such routine (and 64% actually did) is perhaps the most indicative of any health or work benefit. The most commonly reported beneficial effect was an increase in perceived energy levels (including many reported comparisons to the effect of caffeine). The most common discomfortable related adverse reaction was persistent cold sensation in body, hands and/or feet in up to 13% of participants. Other related harmful effects were mild and uncommon.

Influenza was the most common reason for participants’ absenteeism durations longer than five days. This study was performed during the 2014/2015 influenza epidemic in The Netherlands, which lasting a total of 21 weeks had the longest duration since more than 40 years.[17] The influenza-like illness incidence (consulting a general practitioner) was consistently above 10 per 10,000 inhabitants during the study period, with 5 per 10,000 inhabitants representing the threshold for a mild epidemic. An epidemic is defined as an incidence above this threshold for at least two consecutive weeks. The actual incidence of influenza cases was considerably higher, because only a proportion of the patients with influenza-like symptoms consulted the general practitioner. At the start of the season, influenza virus A(H3N2) dominated, while later in the season, influenza virus B was most prevalent. A part of the circulating influenza A-viruses appeared to mismatch with the influenza A-strain in the vaccine. Other prevalent viruses during the study period included the respiratory syncytial virus (RSV), the enterovirus and the rhinovirus.

We searched PubMed, Web of Science, the Cochrane Database of Systematic Reviews, the Cochrane Central Register of Controlled Trials, and Database of Abstracts of Reviews on Effects for articles published between Jan 1, 1980, and Oct 1, 2015 on the effect of any type of cold bathing on health. We used the broad MeSH term “Cold Temperature” in combination with the terms “bath*” or “shower*”. Our search resulted in no randomized controlled trials that assessed health. One Cochrane review investigated the effect of cold-water immersion for preventing and treating muscle soreness after exercise[18] and found some evidence that cold-water immersion reduces delayed onset muscle soreness after exercise. The three randomized controlled trials were limited to the subject of cold bathing on athletic performance[19,20] and physiological response.[3] Positive habituation effects on the physiological response and slight beneficial outcomes on athletic recovery have been reported. However, there is a lack of data regarding any cumulative clinical effect and relevance for health.

Cold water has been used therapeutically for many centuries and continues using modern technology. Hippocrates, the father of medicine, who added rubbing to cold bathing, was accustomed to use cold water in his treatment of the most serious illnesses.[21] Although most cold exposure studies involved cold water immersion, different methods of cold water therapy such as cold bathing and cold showering are used interchangeably and seem to have similar effects.[19] The latest form of cold therapy (or stimulation) is called whole-body cryotherapy and consists of exposure to very cold air that is maintained at -110°C to -140°C in special temperature-controlled cryochambers, generally for 2–3 minutes. It was initially proposed for the treatment of rheumatic diseases[22] but is increasingly popularized among athletes for its supposedly beneficial effect on recovery and performance, even though it has not been confirmed in a recent systematic review.[18,23–24]

In The Netherlands, there has been an increasing trend for cold bathing over the past few years. Part of this growing popularity is owed to the scientific approach of a health and mindset technique hallmarked by cold-exposure as created by an individual named Wim Hof, nicknamed the Iceman for his ability to remain constant body temperature in extreme cold conditions.[25] These methods involving concentration, breathing and cold-exposure have shown to modulate the immune response.[26] These findings served as inspiration to design the present trial and its popularity facilitated recruitment of over three thousand participants in just one month time.

The mechanism or explanatory pathway of any therapeutic effects of cold exposure remains unclear. In the acute phase (during shivering) increases of cortisol and norepinephrine concentrations have been reported but resulted in minimal or no immune modulation.[4–7] Moreover, both immune-stimulatory and immune-inhibiting effects of cold exposure during exercise increase controversy.[27] Data obtained mainly on small mammals suggests that cold exposure suppresses several cellular and humoral components of the immune response but adaptation to a given cold stimulus appears to develop over the course of 2–3 weeks.[28] Beta-endorphin increase has been reported after cold exposure in rats and cold stress-induced modulation of cell immunity has been reported during acute Toxoplasma gondii infection in mice.[29–30] However, these findings could not be reproduced in one study in humans.[31] The current study adds data on cold adaptation following repeated cold exposure. Longterm hormonal and cytokine effects of such modulation are relatively small and its significance remains unclear as only the early steps of the immune cascade appear to be affected.[6] The fact that there was no difference between 30, 60 or 90 seconds of cold showering is consistent with previous research on the habituation of the initial responses to cold water immersion. The greatest physiological response to cold water exposure was observed during the first 30 seconds and the rapidity suggests that it is initiated by neurogenic pathways rather than circulating hormones.[32]

Another physiological explanatory mechanism is the improvement of fitness level when considering the routine cold shower as frequent engaging physical activity. In the present trial, reduction of sickness absence of a routine cold shower (29%) was comparable to the effect of regular physical activity (35%). A previous study in The Netherlands showed that the mean total duration of absenteeism was 15% lower in cyclists than in non-cyclists. Cycling to work was therefore associated with less sickness absence.[16] The more often people cycled to work and the longer the distance travelled, the less they reported sick. This is consistent with the findings of Nieman et al. who have shown in several studies that there is an inverse relationship between physical activity or fitness level and the rates of upper respiratory tract infection.[33] Recently, a meta-analysis of four randomized controlled trials determined the effects of exercise on prevention of the common cold. The effect of exercise on the prevention of the common cold had a relative risk reduction of 27% and there was a mean reduction of 3.5 illness days compared to controls.[34]

In addition, there are multiple psychological explanatory mechanisms such as expectancies which play a major role for the treatment outcome of a broad variety of immune-mediated conditions.[35] The outcome expectations of the present study billed as testing the hypothesis whether “cold-showers-might-decrease-illness-and-improve-health” could potentially play a suggestive role in the actual outcomes such as the decision to go to work when feeling ill. The promotion material included more positive than negative general claims to be explored such as “Habitual cold exposure has been claimed to have positive influences including improvements of the immune system, circulation, emotional state, skin conditions, and energy. The aim of this study is to investigate whether such claims are true.” Other than the statement of these unsupported claims, promotional material did not suggest that cold showers might reduce illness or absenteeism. Prior to the start of the trial, participants were informed of several outcome parameters including vitality, energy levels, work productivity and sickness absence. They were intentionally not informed of primary and secondary outcomes. Participants were fully aware of the four different groups. Other communication forms such as consent form and emails were nonsuggestive. The contrast between the results of both primary endpoints could also suggest that the intervention made participants more resilient to absenteeism with comparable intensity and duration of illness symptoms.

Our data cannot determine whether the present findings were causal or associational. Moreover, participants in this trial could not be blinded for the intervention nor for their own outcome assessment, hence potentially introducing important bias. Specifically, a placebo-effect of this intervention cannot be ruled out. However, if such effect was causative in this trial, it should not be considered as an effect of an “inert substance”.[36] Placebo effects rely on complex neurobiologic pathways involving neurotransmitters such as norepinephrine and activation of specific, quantifiable, and relevant areas of the brain.[37] With the recent discovery of the central nervous system lymphatic system represent, a neurobiologic immunostimulatory effect should not be ruled out.[38]

The findings of this study should be interpreted while accounting for its limitations. First, all outcomes were self-reported based on our online survey design. Hence, none of our parameters could be objectified. Our primary outcome sickness absence was selected for its closest proximity of an objective parameter. Second, according to the SF-36 data, the study population is extremely healthy compared the general Dutch population. This is most likely a correct measurement due to an important sampling bias: (1) all patients with severe comorbidity were excluded; (2) the athletic / strenuous character of the study attracted a highly motivated, healthy and physically active group with SF-36 averages much higher than the population norm though lower than a competitive athletic population;[39] (3) 96% of participants rated their health to be good or excellent; (4) the prevalence of participation in sports (85%) in the study population was higher than the average norm (53% in the national population of 12 years and above);[40] and (5) average sickness absence in the control group (1,55%) was almost a third compared to the average sickness absence in the Dutch population (4,4% during the first quartile of 2015 corresponding to the study period).[41] Third, attrition bias could be introduced because of the large loss to follow-up (20%), which is likely due to the online-only interface of the study. Non-responders could only be contacted by the provided email address. In the intervention groups, large numbers of participants discontinued the intervention because of its burden or a sickness making them choose for their preferred routine. In contrast, in the control group discontinuers were much fewer as the control group instructions to shower as regular did not cause any burden or preference to discontinue due to sickness. Fourth, inherent to the pragmatic design, compliance to the intervention was not verifiable. Participants were asked to record if and how long they continued the intervention. During the first 30 days of the trial the median recorded time of the intervention was equal to the instructed time for each group, which is suggestive of valid measures for dividing the groups. During the last 60 days of the trial the median recorded time for the intervention was different only in the 90s cold shower group (median 60s, interquartile range 10–110). In our opinion there was no incentive to report false data considering the anonymous character of data analysis. Such bias would likely have a tendency to reduce any effect on health and work because of the limited compliance ranging from 64 to 79 percent of participants during the study period. Fifth, the relatively short follow-up period and the very healthy character of the study group resulted in the fact that most participants did not have any sickness absence days at all. Sixth, there was a variation of temperature of the coldest available shower water according to location. However, less cold temperatures would underestimate the effect of the intervention.

The main strengths of this trial include its innovativity, the large number of participants, the randomization to four groups and the pragmatic approach in a domestic setting. It was designed as a straightforward study looking at cumulative subjective effect after a routine behavioral intervention in daily life and significant relevance in terms of effect size. This pragmatic randomized controlled trial is the first study showing that a routine cold shower has a beneficial effect on health.

Repetitive cold showering can modulate the physiological response.[3] Our findings show that routinely showering (hot-to-) cold for at least 30 days resulted in a reduction of self-reported sick leave from work but not illness days in adults without severe comorbidity. Further research using objective parameters is necessary to determine whether these were causal or associational findings. Considering the mild effect of a routine cold shower on hormonal and cytokine modulation, these alone are unlikely to play a significant role.[4–7] Perhaps neuroimaging technologies such as functional MRI could be used to assess any potential neurobiologic immunostimulatory effect.

## Supporting Information

S1 Checklist CONSORT 2010 Checklist. (DOC)

S1 Protocol Protocol version 1 for Institutional Review Board–Dutch version. (PDF)

S2 Protocol Protocol version 1 for Institutional Review Board–English version. (PDF)

S3 Protocol Study Protocol COOL Challenge—definitive version. (DOCX)

S4 Protocol Institutional Review Board approval (including English translation). (PDF)

S1 Table Final model output from SAS PROC GENMOD for sickness absence. (DOCX)

S2 Table Final model output from SAS PROC GENMOD for illness. (DOCX)
