# Correction: The Effect of Cold Showering on Health and Work: A Randomized Controlled Trial

**DOI:** 10.1371/journal.pone.0201978

**Published:** 2018-08-02

**Authors:** Geert A. Buijze, Inger N. Sierevelt, Bas C. J. M. van der Heijden, Marcel G. Dijkgraaf, Monique H. W. Frings-Dresen

The data underlying Table 2 are omitted from the list of Supporting Information. The data can be viewed below.

## Supporting information

S1 File Supplementary dataset. This file includes the data underlying Table 2.
(XLSX)

## References

[1] Buijze GA, Sierevelt IN, van der Heijden BCJM, Dijkgraaf MG, Frings-Dresen MHW (2016) The Effect of Cold Showering on Health and Work: A Randomized Controlled Trial. PLoS ONE 11(9): e0161749 10.1371/journal.pone.0161749 27631616 PMC5025014

