# Supplementary material for: The Effect of Cold Showering on Health and Work: A Randomized Controlled Trial
Source: PLoS One. 2016 Sep 15;11(9):e0161749. doi: 10.1371/journal.pone.0161749 (PMC5025014; doi:10.1371/journal.pone.0161749)
Supplement: S1 Protocol — (PDF) [file pone.0161749.s002.pdf]

Medisch Ethische Toetsings Commissie AMC
t.a.v. het secretariaat
kamer E2-170
Postbus 22660
1100 DD Amsterdam

Datum 27 augustus 2014

Betreft: Aanbieding onderzoeksvoorstel voor beoordeling van de WMO-plichtigheid.

Geachte secretaris,

Hierbij doe ik u toekomen het hieronder genoemde onderzoeksvoorstel ten behoeve van beoordeling van de WMO-plichtigheid door de METC.

Titel: The COOL Challenge: measuring the effect of daily cold exposure on quality of health. A proof-of-concept trial

Hoofdonderzoeker: Prof. Dr. M. Frings-Dresen
Afdeling: Coronel Instituut
Kamernummer: K0-122
Emailadres: m.frings@amc.uva.nl

Uitvoerend onderzoeker: Dr. G.A. Buijze
Emailadres: g.a.buijze@amc.uva.nl

1. Het beoordelingsverzoek is vanwege:
☒ twijfel wel/niet WMO
☐ schriftelijke verklaring i.v.m publicatie
☐ iets anders, nl

2. De onderzoeksvraag luidt: Verbertert een dagelijkse koude douche gedurende 30 dagen de kwaliteit van leven en werk-gerelateerd functioneren?

3. De deelnemers aan het onderzoek zijn: gezonde vrijwilligers (die niet gewend zijn om dagelijks koud (af) te douchen).
Om veiligheidsredenen zullen de vrijwilligers geïnstrueerd worden niet langer dan 90 seconden onder de koude douche te blijven.

.4. Het (geschatte) aantal deelnemers/dossiers is 1000.

5. De werving van de deelnemers zal als volgt verlopen: Via social media (facebook/twitter/mailinglijsten)

6. De toestemming van de proefpersoon zal:
☐ niet gevraagd worden,
☒ schriftelijk gevraagd worden,
☐ mondeling gevraagd worden
Toelichting: Deze proof-of-concept studie heeft een eenvoudige web-based opzet en is alleen uitvoerbaar mits de gezonde proefpersonen zich online kunnen aanmelden en toestemming verlenen om via een website enkele vragenlijsten in te vullen voor en na de periode van 30 dagen.

7. De belasting en risico's voor de deelnemer bij de uitvoering van het onderzoek zijn:

(Denk hierbij aan: (frequentie van) testen, ingrepen, observaties, extra ziekenhuis bezoek, tijdsbelasting, afname lichaamsmateriaal). Er zijn meerdere keuzes mogelijk

☐ retrospectief gegevens worden verzameld en verwerkt (statusonderzoek).
☐ prospectief gegevens worden verzameld en verwerkt.
☐ weefsel wordt gebruikt dat tijdens normale diagnostiek of zorg is verzameld.
☒ 3 keer 2 vragenlijst(en) wordt / worden afgenomen.
☐ eenmalig een urine monster wordt verzameld.
☐ extra bloed wordt afgenomen tijdens reguliere afname.
☐ Anders nl,................

8. Vragenlijsten: (*ter beoordeling van de mogelijkheid dat de psychische integriteit van de proefpersoon wordt aangetast*)
☒ zijn bijgesloten
☐ niet van toepassing

9. De vertrouwelijkheid van de medische en persoonlijke gegevens is als volgt gewaarborgd:
☐ de persoonsgegevens worden gecodeerd en alleen de onderzoeker heeft toegang tot de codesleutel
☒ de onderzoeksgegevens zijn nooit tot de persoon te herleiden *(geanonimiseerd)*

☐ Anders nl,................

10. Beoordeling door een andere erkende METC van dit onderzoek
☐ is bijgesloten
☒ niet van toepassing

11. Beoogde startdatum: 1 november 2014
Beoogde einddatum: 1 april 2015

Met vriendelijke groet,

Dr. G. A. Buijze

AIOS orthopedie en onderzoeker

Bijlages: SF-36 en Health Performance Questionnaire (HPQ) vragenlijsten
