# Supplementary material for: The Effect of Cold Showering on Health and Work: A Randomized Controlled Trial
Source: PLoS One. 2016 Sep 15;11(9):e0161749. doi: 10.1371/journal.pone.0161749 (PMC5025014; doi:10.1371/journal.pone.0161749)
Supplement: S2 Protocol — (PDF) [file pone.0161749.s003.pdf]

Medical Ethical Review Committee AMC
Secretary
Room E2-170
P.O. Box 22660
1100 DD Amsterdam

Date: August 27th 2014

RE: Submission of research protocol for assessment of the Medical Research Involving Human Subjects Act

Dear Secretary,

Herewith I submit our research protocol for assessment of the Medical Research Involving Human Subjects Act that obligates official medical ethical review.

Title: The COOL Challenge: measuring the effect of daily cold exposure on quality of health. A proof-of-concept trial

Primary investigator: Prof.dr. Frings-Dresen
Department: Coronel Institute
Room nr: K0-122
Email: m.fring@amc.uva.nl

Researcher executive: Dr. G.A. Buijze
Email: g.a.buijze@amc.uva.nl

1. This review is for the purpose of:

☒ doubt regarding whether or not the Medical Research Involving Human Subjects Act applies to this study protocol

2. The research question is: What is the effect of a daily cold shower during 30 consecutive days on quality of life and work-related functioning?

3. Research participants are: healthy volunteers (that are not used to shower (hot-to-)cold). For safety reasons, participants will be instructed not to shower cold for longer than 90 seconds.

4. The (estimated) amount of participants/files: +/- 1000

5. Enrolment of participants will be done by use of: social media (twitter,facebook,email)

6. Informed consent procedure:

☐ will not be applied,
☒ written,
☐ oral

Explanation: This proof-of-concept study has a straightforward web-based platform and it can only be performed if volunteers can enroll online and provide online informed consent to participate and fill out questionnaires through a website before and after the period of 30 days.

7. The burden and risks to the participant in the performance of the research are:
   (For example: (frequency of) tests , procedures , observations, extra hospital visits, time load, sampling of body material). There are several options possible:

☐ Retrospective data are collected and processed (file study)

☐ prospective data are collected and processed .
☐ tissue is used that, during normal diagnosis or care has been collected .
☒ 3 times 2 questionaires.
☐ a one-time urine sample.
☐ extra blood will be sampled during regular visits.
☐ Other

8. Questionaires: (to assess the possibility that the psychological integrity of the subject is affected)
☒ are enclosed
☐ not applicable

9. The confidentiality of medical and personal information is safeguarded as follows::
☐ the personal data are encrypted and only the researcher has access to the encryption key
☒ The survey can never be traced back to the person (anonymous)
☐ Other.

10. Review by another accredited IRB of this research
☐ is attached
☒ not applicable

11. Expected start date: November 1st 2014
Expected end date: April 1st 2015

Sincerely,

Dr. Geert A. Buijze

Orthopedic Resident and Researcher

Attachments: SF-36 and Health Perfomance Questionaire (HPQ)
