# Supplementary material for: The Effect of Cold Showering on Health and Work: A Randomized Controlled Trial
Source: PLoS One. 2016 Sep 15;11(9):e0161749. doi: 10.1371/journal.pone.0161749 (PMC5025014; doi:10.1371/journal.pone.0161749)
Supplement: S3 Protocol — (DOCX) [file pone.0161749.s004.docx]

**S3 Protocol**

COOL Challenge 2015

The effect of a daily cold shower on health and work:

A proof-of-concept trial

*Dr. G.A. Buijze, Dr. B. Bakker, K. De Jong; M.G.Dijkgraaf; Prof. dr. M.H.W. Frings-Dresen*

INTRODUCTION

Is our autoimmune system weakening with the generations? This worrisome trending topic of debate is arguable in terms of empirical evidence base. Hypotheses in its support include contemporary artificial standards of living which the immune system is stimulated as much constant acclimatization and excessive hygiene.^1^ An illustrative example is the health trend of pharmacological immune boosting, e.g. millions of Europeans routinely take daily vitamin supplements and / or probiotics in the hope of improving their immune system: a controversial billion dollar industry despite limited evidence.^2^

A more important question would be: Are we able to strengthen our autoimmune system physiologically ? Exposure to cold by immersion in cold water or cryotherapy (freezing cold air flow) is effective for short duration as it generates strong reactions in the autonomic nervous system and metabolism by increase in beta - endorphins and noradrenaline. ^3-5^ Repetitive cold exposure on the long run seems to have some positive effect on the autoimmune system and is being studied as add-on therapy for rheumatism. ^6^ Recently it was conclusively shown that the method of Wim Hof ​​- where cold exposure is central – has a highly significant influence on the autoimmuunsysteem.^7^ Whether repetitive cold exposure can actually improve the immune system significantly remains a point of discussion in lack of high quality research.

Strengthening the immune system has (besides a great social benefit) an important economic advantage. According to the Central Bureau for Statistics (CBS) absenteeism is highest in the first quarter of the year with an average of 4.5 %. That means daily one of the 22 employees are on sick leave.^8^ Even a slight improvement has already a significant macroeconomic impact.

It is therefore of great scientific interest to conduct an adequately powered proof-of-concept trial : The COOL Challenge.

AIM

The primary aim is to determine whether a regular (hot-to-) cold shower during 30 consecutive days can have a beneficial effect on immune function by reducing sickness absence and related illness. Secondary aims are to determine effects on quality of health and work productivity.

METHODS

INCLUSION CRITERIA

-Men and women aged 18-65

-Employed

-No previous experience of cold bathing

EXCLUSION CRITERIA

-Significant comorbidity (such as severe cardiovascular or pulmonary disease)

INTERVENTIONS

Daily cold shower during 30 days starting warm as hot and long as preferred but ending with 30, 60 or 90 seconds according to group showering at the coldest available temperature

Intervention period : January 1st-30th 2015.

Follow-up period: January 31st-March 31st 2015. Participants of all three intervention groups are instructed to shower cold or warm as preferred

PRIMARY OUTCOME Sickness absence in study period (from January to March 2015)

SECONDARY OUTCOMES

Quality of health (SF-36 and BSI) and work productivity (UWES)

Adverse events and experience

Measurements: Website (online questionnaires/database) www.coolchallenge.nl

PROTOCOL

**Enrolment:** Screening with questionnaires via online registration followed by randomisation (01 dec – 31 dec 2014). Volunteers will be contacted by email.

**Experimental group:** 1– 30 jan 2015 once daily (hot-to-)cold shower (10-12°C) during exactly 30, 60 or 90 seconds. Hot shower before as preferred. Additional daily normal (hot) shower (>20°C) is allowed.

**Control group:** 1– 30 jan 2015 habitual (hot) shower (>20°C) as preferred. Cold shower not permitted.

**Outcome assessment**: By email with a link to the online Survey: After inclusion, 30 and 90 days.

**Cross-over:** Allowed after 30 days for intervention group, analysis using ITT principes

**Follow-up:** 1 jan - 31 maart 2015.

STATISTICS

575 individuals will achieve 80% power (with alpha=0.05) to detect a difference of 0.5 days of sickness absence (SD 3.03) during the 90 days period, based on previous data.^9^

Accounting for a 20% lost to FU, target enrolment at 720 individuals.

ORGANISING/RESEARCH TEAM

Dr. Geert Buijze (Executive Investigator)

Koen de Jong (Physiologist)

Linda Koeman (Logistics)

Klaas Kroezen (Web/Online Expert)

Dr. Bram Bakker (Coach & Psychiatrist)

Prof. dr. M.H.W. Frings-Dresen (Principal Investigator, Coronel Instituut)

Dr. M.G.W. Dijkgraaf (Clinical Epidemiologist / Methodologist / IRB Member)

REFERENCES

1. Shevchuk NA. Adapted cold shower as a potential treatment for depression. Med Hypotheses. 2008;70(5):995-1001. Epub 2007 Nov 13.

2. Sinvani LD, et al. Vitamins and Dietary Supplements: Controversy and Confusion. Clinical Geriatrics: 2013 May; 21(5).

3. Castellani JW1, M Brenner IK, Rhind SG. Cold exposure: human immune responses and intracellular cytokine expression. Med Sci Sports Exerc. 2002 Dec;34(12):2013-20.

4. Janský L1, Pospísilová D, Honzová S, Ulicný B, Srámek P, Zeman V, Kamínková J. Immune system of cold-exposed and cold-adapted humans. Eur J Appl Physiol Occup Physiol. 1996;72(5-6):445-50.

5. SÏramek P, SÏimecÏkova M. Jansky L, SÏavlõkova J, Vybõra S. Human physiological responses to immersion into water of different temperatures. Eur J Appl Physiol (2000) 81: 436-442.

6. Jastrząbek R1, Straburzyńska-Lupa A, Rutkowski R, Romanowski W. Effects of different local cryotherapies on systemic levels of TNF-α, IL-6, and clinical parameters in active rheumatoid arthritis. Rheumatol Int. 2013 Aug;33(8):2053-60.

7. Kox M, van Eijk LT, Zwaag J, van den Wildenberg J, Sweep FC, van der Hoeven JG, Pickkers P. Voluntary activation of the sympathetic nervous system and attenuation of the innate immune response in humans. Proc Natl Acad Sci U S A. 2014 May 20;111(20):7379-84.

8. Centraal Bureau voor Statistiek. http://www.cbs.nl/nl-NL/menu/themas/arbeid-sociale-zekerheid/publicaties/arbeidsmarkt-vogelvlucht/korte-termijn-ontw/2006-arbeidsmarkt-vv-ao-zv-art.htm, accessed May 2^nd^ 2014.

9. Hendriksen IJ, Simons M, Garre FG, Hildebrandt VH. The association between commuter cycling and sickness absence. Prev Med. 2010;51:132-5.
