# Supplementary material for: The Effect of Cold Showering on Health and Work: A Randomized Controlled Trial
Source: PLoS One. 2016 Sep 15;11(9):e0161749. doi: 10.1371/journal.pone.0161749 (PMC5025014; doi:10.1371/journal.pone.0161749)
Supplement: S4 Protocol — (PDF) [file pone.0161749.s005.pdf]

Academisch Medisch Centrum
Universiteit van Amsterdam

Aan mevrouw prof.dr. M.H.W. Frings-Dresen
Coronel Instituut
K0-122

Amsterdam, 3 september 2014
uw kenmerk:
ons kenmerk: W14_244 # 14.17.0296
betreft:

Medisch Ethische Toetsingscommissie
E2-170
doorkiesnummer: 566 7389

Uw brief: **The COOL Challenge: measuring the effect of daily cold exposure on quality of health. A proof-of-concept trial**

Geachte mevrouw Frings-Dresen,

Uw brief d.d. 27 augustus 2014 betreffende bovengenoemde studie is op 2 september jl. besproken in de vergadering van het dagelijks bestuur.

Het betreft een onderzoek met gezonde vrijwilligers naar de vraag of een dagelijkse koude douche gedurende 30 dagen de kwaliteit van leven en het werk-gerelateerd functioneren verbetert.

Het dagelijks bestuur is van oordeel dat bovengenoemde studie niet valt binnen de reikwijdte van de Wet medisch-wetenschappelijk onderzoek met mensen, aangezien er geen sprake is van wetenschappelijk onderzoek zoals bedoeld in artikel 1, eerste lid onder b van de WMO, daar er geen sprake is van een medisch-wetenschappelijk onderzoek, omdat er geen sprake is van een medisch-wetenschappelijke vraagstelling.

Een formele beoordeling door onze commissie is derhalve niet noodzakelijk.

Met vriendelijke groet,
namens de Medisch Ethische Toetsingscommissie

Mw. T. Groenveld,
ambtelijk secretaris

Bijlage: verklaring in het Engels (z.o.z.)

c.c. per email: g.a.buijze@amc.uva.nl

Meibergdreef 9 Postbus 22660 1100 DD Amsterdam T 020 5669111 F 020 5664440 www.amc.nl

To whom it may concern,

Referring to our letter of September 3rd, 2014 (reference number W14_244 # 14.17.0296) we are pleased to confirm that the Medical Research Involving Human Subjects Act (WMO) does not apply to the above mentioned study and that an official approval of this study by our committee is not required.

Yours sincerely,
on behalf of the Medical Ethics Review Committee,

Mrs. T. Groenveld
secretary
