# Supplementary material for: The Effect of Cold Showering on Health and Work: A Randomized Controlled Trial
Source: PLoS One. 2016 Sep 15;11(9):e0161749. doi: 10.1371/journal.pone.0161749 (PMC5025014; doi:10.1371/journal.pone.0161749)
Supplement: S1 Table — (DOCX) [file pone.0161749.s006.docx]

**S1 Table.** Final model output from SAS PROC GENMOD for sickness absence.


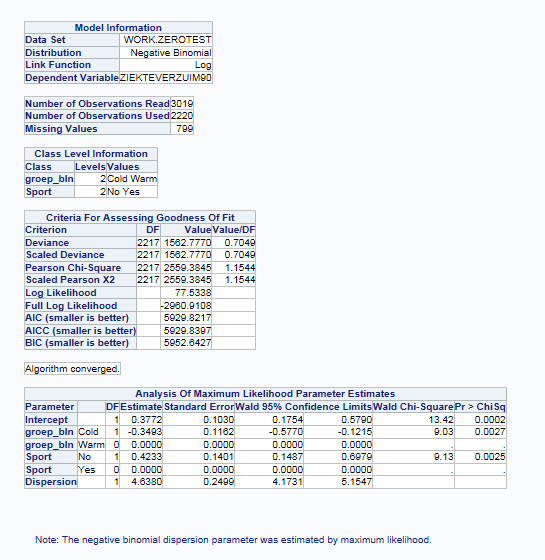


**Table 5. Final model output from SAS PROC GENMOD for sickness absence:**

**Table 5. Final model output from SAS PROC GENMOD for sickness absence:**

**Table 5. Final model output from SAS PROC GENMOD for sickness absence:**

**Table 5. Final model output from SAS PROC GENMOD for sickness absence:**

This study investigated the effect of cold showering on health and work: a trial randomizing a (hot-to-) cold shower for 30, 60, 90 seconds or a control group during 30 consecutive days followed by 60 days of showering cold at their own discretion for the intervention groups.
